# Mistaking Covariance for Combination in Sensorimotor Adaptation: Regression Slopes Do Not Test Additivity

**DOI:** 10.1523/ENEURO.0474-25.2026

**Published:** 2026-05-19

**Authors:** Joshua Liddy

**Affiliations:** Department of Kinesiology, University of Massachusetts Amherst, Amherst, Massachusetts 01003

**Keywords:** additivity, explicit strategy, implicit recalibration, model assumptions, motor learning, sensorimotor adaptation

## Abstract

Sensorimotor adaptation depends on implicit recalibration and explicit strategy. These processes are commonly assumed to sum (*A* = *I* + *E*), and this additivity assumption justifies subtractive measurement and informs computational models of motor learning. Recent work has challenged additivity by examining regression slopes between implicit and explicit measures. When slopes deviate from *β* = −1, the interpretation has been that the processes are “sub-additive” and fail to sum as expected. Here, we show this reasoning is mistaken. Regression slopes reflect covariance structure: how learning processes relate across individuals. Additivity is a claim about motor output combination: whether learning processes sum within individuals. These are different questions, and regression slopes do not address the latter. We derive the expected slope under subtractive logic and show it equals *β* = −1 only when total adaptation is uncorrelated with the measured component. Monte Carlo simulations confirm this benchmark is routinely rejected under realistic covariance structures, even when additivity is enforced. Under independent measurement of the learning processes, the regression slope depends on covariance structure, in which additivity does not constrain. Thus, there is no regression slope benchmark for diagnosing additivity. Moreover, the regression slopes reported in previous studies fall within the range predicted by shared-error models that adhere to the additivity assumption. Regression slopes do not test additivity; they only indicate how implicit and explicit learning covary across individuals. Challenging the additivity assumption will require direct tests of motor output combination and formal model comparison.

## Significance Statement

When learning or refining motor skills, the brain uses both conscious strategies and unconscious adjustments. Researchers have long assumed that these processes sum to produce behavior. This assumption justifies standard measurement techniques and models of motor learning. Recent studies claim to invalidate this assumption, which would require the field to revise current approaches. We show that the statistical test behind this claim is flawed because it asks whether people who rely more on one process rely less on the other, not whether the learning processes sum. The question of whether motor learning processes combine additively remains unresolved. Resolving this issue will require direct tests of motor output combination and formal model comparison.

## Introduction

Sensorimotor adaptation depends on at least two learning processes—implicit recalibration and explicit strategy—that are assumed to sum when producing motor output (*A* = *I* + *E*; Smith et al., 2006; Taylor et al., 2014). This is not merely a convenient simplification; it serves as the foundation for a large body of literature in which learning processes are estimated using subtractive logic (*I *= *A *− *E*; Bond and Taylor, 2015; McDougle et al., 2015; Werner et al., 2015; Neville and Cressman, 2018; Modchalingam et al., 2019, 2023; Miyamoto et al., 2020; Vachon et al., 2020; Wilterson and Taylor, 2021; Albert et al., 2022). Theoretically, it is consistent with a modular architecture in which learning processes combine at expression, independent of how they interact at acquisition. Whether this assumption is valid determines whether current computational models need to be reconsidered.

The additivity assumption has been challenged by analyses suggesting that the relationship between implicit and explicit learning is “sub-additive.” Specifically, ‘t Hart et al. (2024) argued that if additivity holds, the regression slope of implicit recalibration on explicit strategy must be −1. Because empirical slopes deviated significantly from this value, they concluded that additivity was violated. Similarly, Chen and Taylor (2025) recently reported “sub-additive” slopes even when obtaining independent measures of implicit and explicit processes, interpreting this as evidence for more complex interactions among learning systems.

If correct, this would be a significant finding, requiring the field to abandon subtractive logic and develop new models of how learning processes combine. But the evidence does not support this conclusion. We argue that the apparent rejection of additivity rests on a category error. Additivity is a claim about combination: whether learning processes sum to produce motor output within individuals. Regression slopes reflect covariance structure: how those processes relate across individuals. These are different questions.

We show that, under subtractive logic, regression slopes are analytically determined by the covariance between total adaptation and the measured component, not by whether additivity holds. We then show that independent measurement does not solve this problem, because the covariance structure of implicit and explicit processes is not constrained by additivity. Finally, we argue that the accumulating reports of “sub-additivity” are compatible with shared-error models (Miyamoto et al., 2020; Albert et al., 2022), in which explicit system reduces the error available to the implicit system.

Thus, regression slopes do not test whether learning processes sum within individuals, only how those processes covary across individuals. The *β* = −1 benchmark is one instance of this error, but the problem is more general: between-subject regression slopes cannot diagnose additivity. Additivity may not be a valid assumption, but answering that question will require approaches that directly address combination rather than covariance.

## What Is Additivity?

Additivity is the assumption that implicit and explicit processes sum to produce motor output:

A=I+E.

This is a claim about combination. The combination rule is linear rather than multiplicative, gated, or winner-take-all. It says that whatever values *I* and *E* take, they sum to produce *A*. How those values are acquired—whether through independent learning (Taylor and Ivry, 2011), shared-error signals (Albert et al., 2022), or mutual compensation (Miyamoto et al., 2020)—is not specified.

In sensorimotor adaptation, we further assume unit weights such that each learning process contributes its full value to the motor output. This does not mean the processes contribute equal amounts, only that a one-unit change in either *I* or *E* produces an equivalent change in *A*. This is convenient, but not necessary. A more general additive model could allow:

$$A=w_{I}\cdot I+w_{E}\cdot E,$$

where the weights could differ from unity, from each other, or across individuals. For now, we follow standard convention, but nothing in our argument depends on this choice.

Under subtractive logic, where implicit adaptation is computed as *I* = *A* − *E*, the identity *A* = *I* + *E* holds by definition—it is an algebraic identity, not a hypothesis. This construction also induces mathematical coupling: *E* appears on both sides because *I* contains −*E*, so any *I* − *E* association is partly structural and will reflect covariance in *A* and *E* rather than the combination rule. The additivity assumption becomes testable only when all three quantities are measured independently. In that case, *A* = *I* + *E* is an empirical claim that three separately measured quantities sum.

What does additivity not imply? Additivity does not require independent learning: there are no constraints on how the processes acquire their values. For example, they may learn from the same error signal (Albert et al., 2022). Additivity does not require process independence: two variables can sum to a third while being positively correlated, negatively correlated, or uncorrelated (Miyamoto et al., 2020; Albert et al., 2022). Additivity does not require neural separation: it is a claim about computational combination, not neural implementation. The processes could be instantiated in overlapping circuits or mutually inhibit each other during learning, so long as their contributions sum.

**Figure 1.**
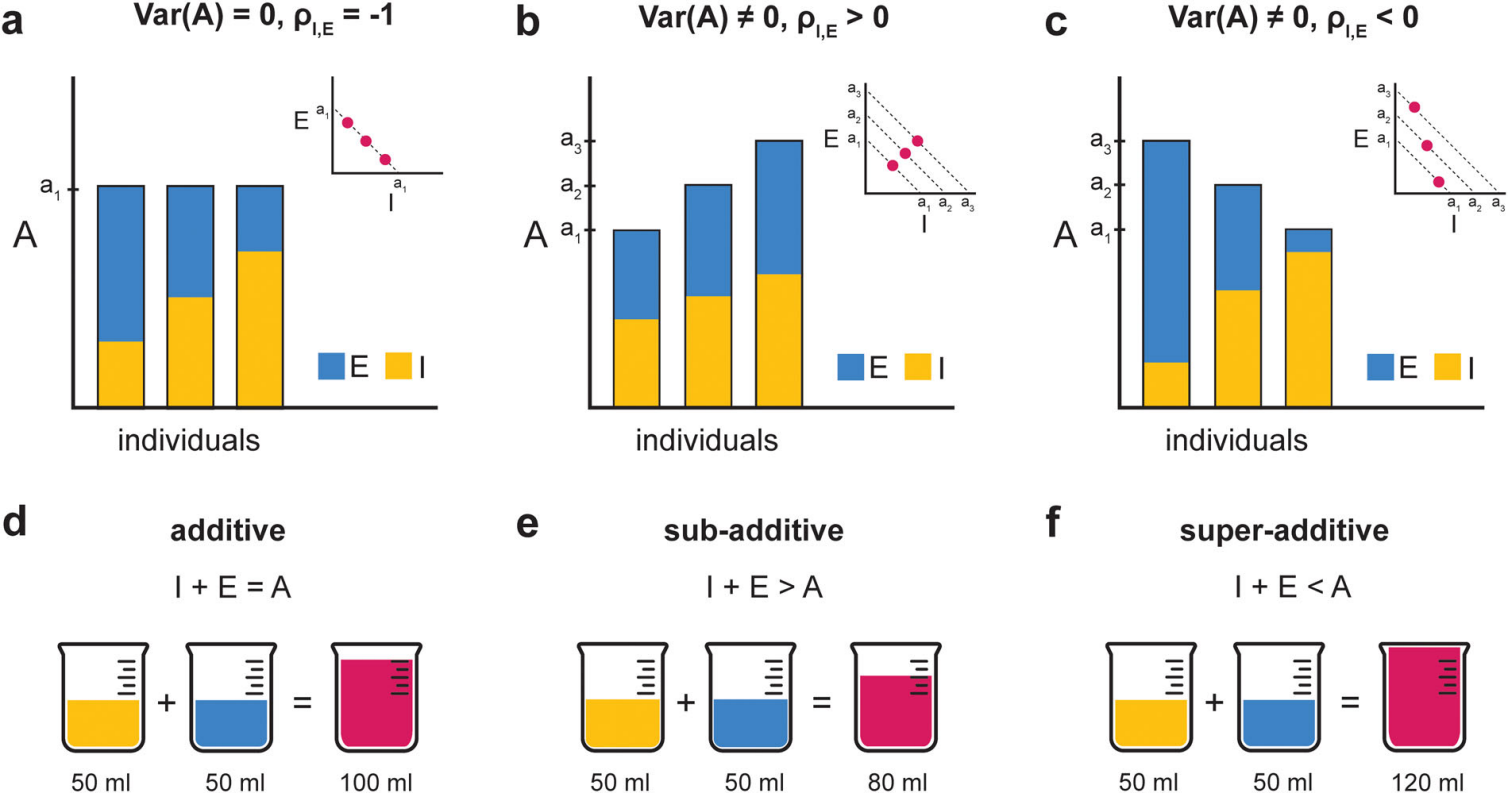
Covariance structure is independent of additivity. ***a–c***, Stacked bars show implicit (*I*, yellow) and explicit (*E*, blue) contributions for three individuals with bar height equal to total adaptation (*A* = *I* + *E*, red). Insets plot *I* against *E*; dashed lines indicate iso-A contours. ***a***, A is fixed with different proportions: bars are of equal height, all points fall on a single iso-*A* line with a correlation of −1. ***b***, A varies with proportions fixed: larger bars have more of both components, so points cross iso-*A* lines with a positive correlation of 1. ***c***, A varies and proportions shift: points span multiple iso-*A* lines while retaining a negative correlation of −1. Additivity holds in all panels, but when *A* varies, the correlation can range from −1 to +1. ***d–f***, Non-additivity requires independent measurement before combination. ***d***, Additive: the parts sum to the total. ***e***, Sub-additive: the total is less than the sum of its parts. ***f***, Super-additive: the total is greater than the sum of its parts.

Importantly, additivity constrains the sum, not the covariance between learning processes. Figure 1, *a*–*c*, shows three populations where *I* + *E* = *A* is true for every individual (stacked bars). Despite adhering to additivity, the correlation between *I* and *E* varies from perfectly negative (Fig. 1*a*) to positive (Fig. 1*b*) to negative again (Fig. 1*c*). The critical difference is whether total adaptation is constant or varies across individuals.

Figure 1, *d*–*f*, clarifies what non-additivity implies. If implicit and explicit processes were measured independently and then combined, additivity predicts the sum equals total adaptation (Fig. 1*d*). Sub-additivity would mean the combination yields less than the sum of its parts (Fig. 1*e*), whereas super-additivity would mean it yields more (Fig. 1*f*). Importantly, additivity is falsifiable: if motor output were non-additive, independent measures would reveal systematic deviations from *A* = *I* + *E*. Detecting non-additivity requires independent measurement with sufficient precision, where what counts as sufficient depends on the expected loss or gain in combination. The key question, then, is whether regression slopes diagnose additivity. Next, we show that they do not.

## The Slope Test Rejects Additivity Even When It Is Enforced

Recently, ‘t Hart et al. (2024) proposed that deviations from a regression slope of −1 in the relationship between implicit and explicit learning indicate a violation of additivity. The logic seems intuitive: if the processes sum and total adaptation is fixed, an inverse tradeoff is obligatory. This benchmark, however, is not diagnostic of additivity.

To clarify why, we derive the expected regression slope between implicit and explicit learning under an additive model. This derivation shows that the slope depends on the covariance between *A* and *E* (or *I*), not on additivity alone. Here, we use subtractive logic consistent with aim reporting procedures (Taylor et al., 2014), where *A* and *E* are observed and *I* is derived. The implications are the same for process dissociation procedures (Werner et al., 2015), which rely on the same subtractive logic in reverse (*E* = *A* − *I*).

Suppose the additive model holds: *A* = *I* + *E*. Rearranging terms gives the algebraic identity: *I* = *A* − *E*. This seems to suggest that the regression slope of *I *– *E* should equal −1, indicating a perfect tradeoff. However, regression coefficients reflect statistical relationships, not algebraic identities. The regression slope, *β*, is defined as the ratio of the covariance between predictor and outcome to the variance of the predictor:

$$\beta =\frac{\mathrm{Cov}(I,E)}{\mathrm{Var}(E)}.$$

Substituting the additive identity *I* = *A* − *E*:

Cov(I,E)=Cov(A−E,E)=Cov(A,E)−Var(E).

Substituting this back into the slope definition allows us to simplify the expression:

$$\beta =\frac{\mathrm{Cov}(A,E)-\mathrm{Var}(E)}{\mathrm{Var}(E)}=\frac{\mathrm{Cov}(A,E)}{\mathrm{Var}(E)}-1.$$

To make the dependency on correlation apparent, we can express the covariance as

$$\mathrm{Cov}(A,E)=\rho_{A,E}\cdot \sigma_{A}\cdot \sigma_{E},$$

where $\rho_{A,E}$ is the correlation coefficient and σ represents the standard deviation:

$$\beta =\rho_{A,E}\frac{\sigma_{A}}{\sigma_{E}}-1.$$

What follows from this? Under subtractive logic, additivity is true by construction, so it cannot be tested this way. This applies whether *I* is derived from *A* and *E* (aim reporting) or *E* is derived from *A* and *I* (process dissociation). The *β* = −1 benchmark implies a perfect tradeoff: every degree of explicit strategy “costs” exactly one degree of implicit recalibration. But this tradeoff is only forced when total adaptation is constant across individuals (Albert et al., 2022, Appendix 3). If everyone adapts the same amount, then variation in *E* must be offset by *I*.

Total adaptation is never constant, and this variance breaks the forced tradeoff. When *A* varies, the slope depends on how that variance relates to *E* (or *I*), not on whether the components sum. One might look for a more context-dependent benchmark, perhaps derived from the observed covariance in a given dataset. But that does not solve the problem. The regression slope is entirely determined by the covariance between *A* and *E* (or *I*), and additivity places no constraint on that quantity. Different covariance structures therefore produce different slopes, all consistent with *A* = *I* + *E*. For this reason, “sub-additive” is a misnomer because *A* = *I* + *E* by construction. A slope shallower than −1 means that people who adapt more tend to use more explicit strategy.

To verify that this analytical result holds under realistic conditions, we conducted a Monte Carlo simulation using values approximating those reported for the aiming group in ‘t Hart et al. (2024): *n* = 25, *μ*_A_ = 25°, *σ*_A_ = 2.5°, *μ*_E_ = 5°, *σ*_E_ = 2.5°. With equal standard deviations, the expected slope simplifies to *β* = *ρ*_A,E _− 1, though our argument does not depend on this simplification. For each level of *ρ*_A,E_ from −0.9 to 0.9, we generated 10,000 datasets by sampling *A* and *E* from a bivariate normal distribution and computing *I* = *A* − *E*. We then fit a linear regression (*I* ∼ *E*) and recorded the slope and 95% confidence interval.

**Figure 2.**
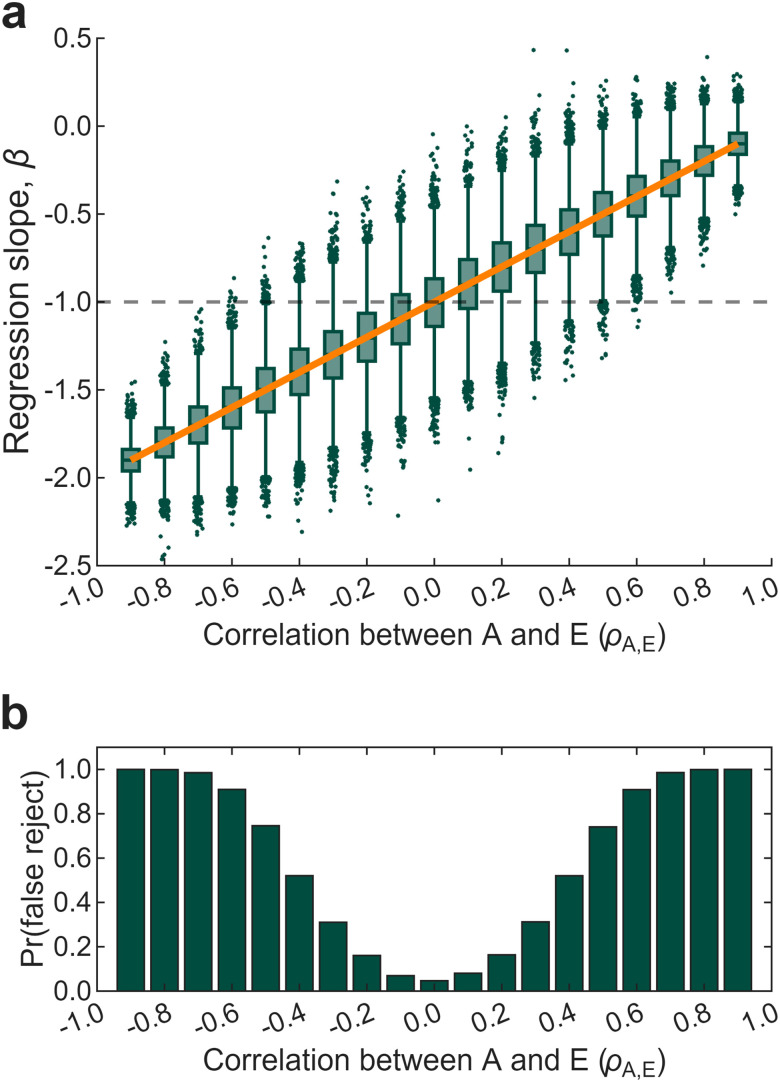
The *β* = −1 benchmark requires constant total adaptation. Data were simulated under subtractive logic (*I* = *A* − *E*), where additivity holds by construction. ***a***, Regression slopes vary systematically with *ρ*_A,E_. The orange line shows the expected slope (*β* = *ρ* − 1). The dashed line indicates the *β* = −1 benchmark. ***b***, Rejection rates for the *β* = −1 benchmark as a function of *ρ*_A,E_. At *ρ*_A,E_ = 0, rejection matches the nominal 5% level. As |*ρ*_A,E_| increases, rejection approaches 100%.

As predicted by Equation 7, the observed slopes tracked the predicted slope exactly (Fig. 2*a*). The *β* = −1 benchmark was recovered only when *ρ*_A,E_ = 0. At all other correlations, the test was unreliable even though the identity *A* = *I* + *E* was true by construction. False rejection rates exceeded 50% at |*ρ*| > 0.4 and approached 100% at |*ρ*| > 0.8 (Fig. 2*b*). Even if the regression slope could test additivity, its inferential reliability would be poor.

In summary, conducting regression on subtraction-derived measures does not test additivity. Under subtractive measurement, the slope reflects covariance structure: how total adaptation relates to the measured component. A slope of −1 indicates zero covariance; a shallower slope indicates positive covariance. Neither tells us whether *A* = *I* + *E*. The “sub-additive” slopes reported in the literature show that people who adapt more tend to rely more explicit strategy than implicit recalibration. That is a statement about individual differences in motor learning, rather than a violation of additivity.

## Independent Measurement Does Not Rescue the Slope Test

One reasonable objection is that the preceding analysis applies only to subtractive methods. If you want to test whether *A* = *I* + *E*, you should measure all three quantities independently. This is sound methodological practice, and previous work has moved in this direction. ‘t Hart et al. (2024) and Chen and Taylor (2025) measured implicit recalibration (exclusion trials) and explicit strategy (aim reports) independently, avoiding the circularity of subtractive approaches.

But independent measures are necessary, not sufficient. The slope test encounters a different problem with the same result. When *I* and *E* are measured independently, the expected regression slope is:

$$\beta =\rho_{I,E}\frac{\sigma_{I}}{\sigma_{E}}.$$

Thus, the slope is determined entirely by how implicit and explicit learning covary across individuals and by their relative variance. A slope of 0 is expected only when *ρ*_I,E_ = 0. More importantly, additivity places no constraint on this covariance; it only claims that *A* = *I* + *E* within individuals. Implicit and explicit learning can sum perfectly while being positively correlated, negatively correlated, or uncorrelated. All three scenarios satisfy *A* = *I* + *E*, meaning there is no slope that confirms additivity and no slope that refutes it.

To verify this, we simulated independent measurement under an additive model. We sampled *I* and *E* from a bivariate normal distribution (*n* = 25, 10,000 iterations per condition) across three variance ratios: *σ*_I_/*σ*_E_ = 0.5, 1.0, and 2.0. For each level of *ρ*_I,E_ from −0.9 to 0.9, we then regressed *I* ∼ *E* and recorded the slope and 95% confidence interval.

**Figure 3.**
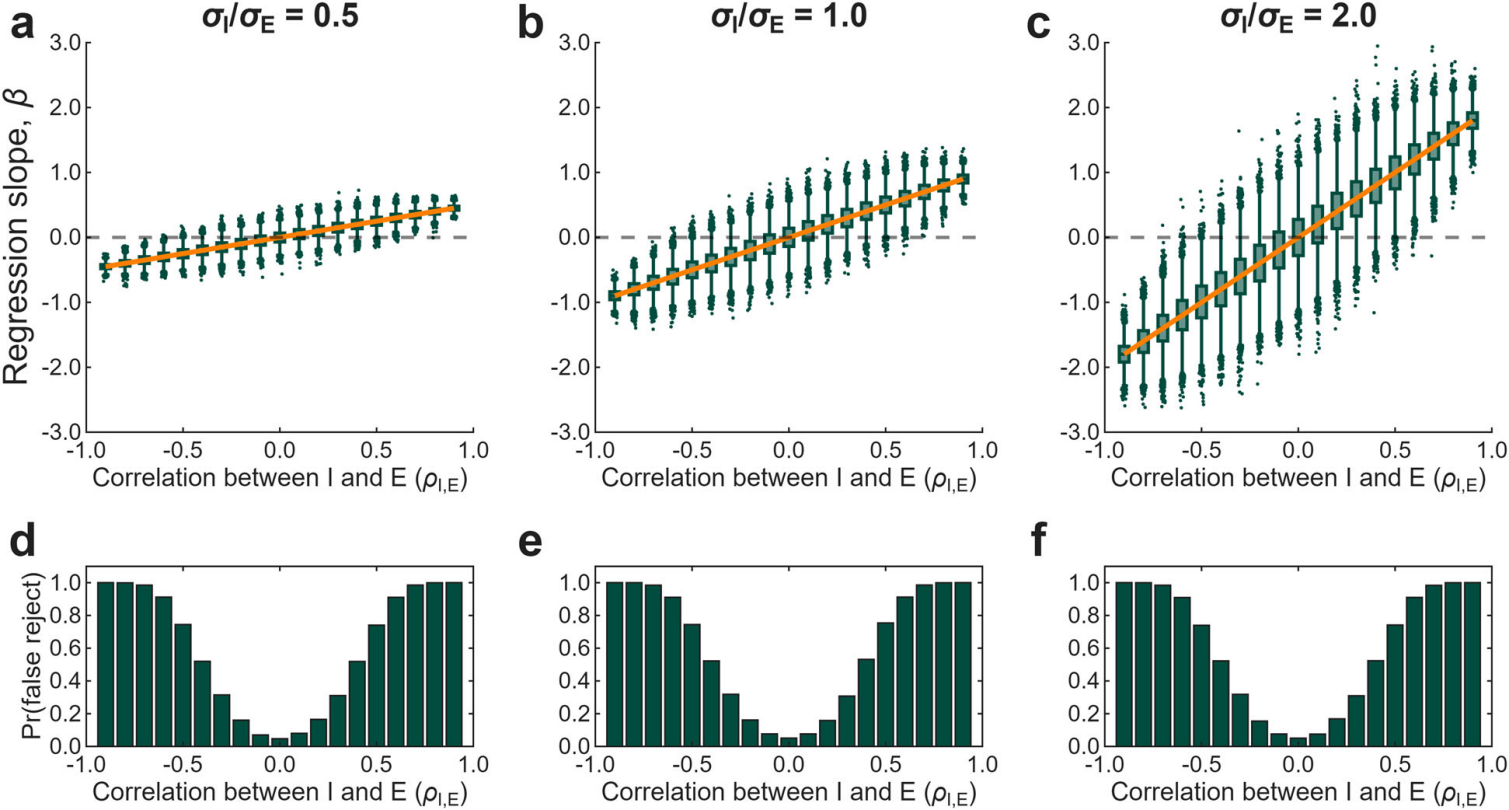
Under independent measurement, regression slopes reflect covariance structure. We sampled *I* and *E* from a bivariate normal distribution (*n* = 25, 10,000 iterations per condition). Columns show three variance ratios: *σ*_I_/*σ*_E_ = 0.5, 1.0, and 2.0. ***a–c***, Regression slopes vary systematically with *ρ*_I,E_. The orange line shows the expected slope (*β* = *ρ*_I,E_ × *σ*_I_/*σ*_E_). The dashed line indicates *β* = 0, the expected value when the learning processes are uncorrelated. ***d–f***, Rejection rates for the *β* = 0 benchmark as a function of *ρ*_I,E_. When *ρ*_I,E_ = 0, rejection rates match the nominal 5% level. As |*ρ*_I,E_| increases, slopes increasingly deviate from zero. When *σ*_I_ < *σ*_E_, slopes are compressed toward zero even with strong negative correlations.

As predicted by Equation 8, the observed slopes tracked the expected values exactly (Fig. 3*a–c*). When *ρ*_I,E_ = 0, slopes clustered around zero. When *σ*_I_ < *σ*_E_, slopes were compressed toward zero. For *σ*_I_/*σ*_E_ = 0.5, a correlation of −0.6 produced a slope of −0.3. This means moderate slopes do not imply weak correlations. In some cases, they may reflect strong correlations attenuated by unequal variance among learning processes.

For example, Chen and Taylor (2025) independently measured implicit recalibration and explicit strategy. Yet they compared their observed slopes to *β* = −1, a benchmark derived by simulating *I* = *A* − *E*. This is a mismatch, because the subtractive benchmark does not apply to independent measures. Under independent measurement, the expected slope is *ρ*_I,E_ scaled by the variance ratio (Eq. 8). But this is beside the point. There is no benchmark comparison because additivity places no constraint on this correlation.

Regression is the wrong test. It quantifies how measured variables covary across a sample, which is useful for asking whether people who show more of one learning process show less of another. But additivity is not a claim about covariance. It is a claim about combination: within each person, do the learning processes sum? We agree that independent measures are preferable, but even then, testing the *I* ∼ *E* slope does not provide evidence against additivity.

## “Sub-additive” Slopes Are Predicted by Additive Shared-Error Models

While regression slopes do not diagnose additivity, they remain valid measures of the statistical relationship between learning processes. The empirical slopes reported in recent studies (‘t Hart et al., 2024; Chen and Taylor, 2025) indicate a near zero to moderately negative relationship between implicit recalibration and explicit strategy use. Importantly, this does not imply weak correlation, because slope estimates reflect correlation scaled by the variance ratio (*σ*_I_/*σ*_E_).

The question is whether these slopes are anomalous or expected. Shared-error coupling models predict that they are expected. ‘t Hart et al. (2024) and Chen and Taylor (2025) interpreted these slopes as evidence against additivity. But the reported negative relationships are predicted by additive models where implicit and explicit learning are coupled through a shared-error signal (Miyamoto et al., 2020; Albert et al., 2022).

In the shared-error model (Albert et al., 2022), implicit recalibration responds to the residual error remaining after explicit strategy is accounted for: *I* = *p*_i_(*r *− *E*), where *p*_i_ reflects implicit learning capacity at steady state, which is jointly determined by its retention and error sensitivity. This is an additive model because total adaptation is the sum of the two learning components.

Albert et al. (2022; Appendix 7) derive three predictions that bear directly on the expected relationships among learning measures. First, competition between *I* and *E* produces a negative relationship because the implicit process can only respond to residual error. Larger explicit strategies leave smaller errors, yielding less implicit recalibration.

Second, explicit strategy and total adaptation should be positively related. Because implicit learning only partially compensates for residual error (*p*_i_ < 1), each additional degree of explicit strategy “costs” less than one degree of implicit learning. The net effect is that individuals who employ larger strategies achieve greater total adaptation.

Third, implicit recalibration and total adaptation will be negatively related when implicit learning is constant across individuals. Under this constraint, the only way to achieve high implicit recalibration is through low explicit strategy, which leaves more residual error to drive the implicit system. When implicit learning varies across individuals, this relationship weakens or even reverses (Albert et al., 2022; Appendix 8).

To show that the shared-error model reproduces empirical slope estimates, we simulated a population of 1,000 participants adapting to a 30° rotation. For each simulated participant, explicit strategy was sampled from a normal distribution (*μ* = 12°, *σ* = 4°). Implicit adaptation was computed from the residual error relation: *I* = *p*_i_(*r* − *E*), with *p*_i_ = 0.65, consistent with empirical estimates (Albert et al., 2022). Total adaptation was defined as *A* = *I* + *E*.

To simulate realistic measurement conditions, we added independent Gaussian noise to implicit (*σ* = 2°) and explicit (*σ* = 4°) measures, reflecting the greater variability commonly observed in aim reports. We then computed regression slopes for all three pairwise relationships (*I* ∼ *E*, *E* ∼ *A*, *I* ∼ *A*). To characterize sampling variability, we bootstrapped 10,000 samples of *n* = 25 (matching typical sample sizes) and computed the distribution of regression slopes for each relationship.

**Figure 4.**
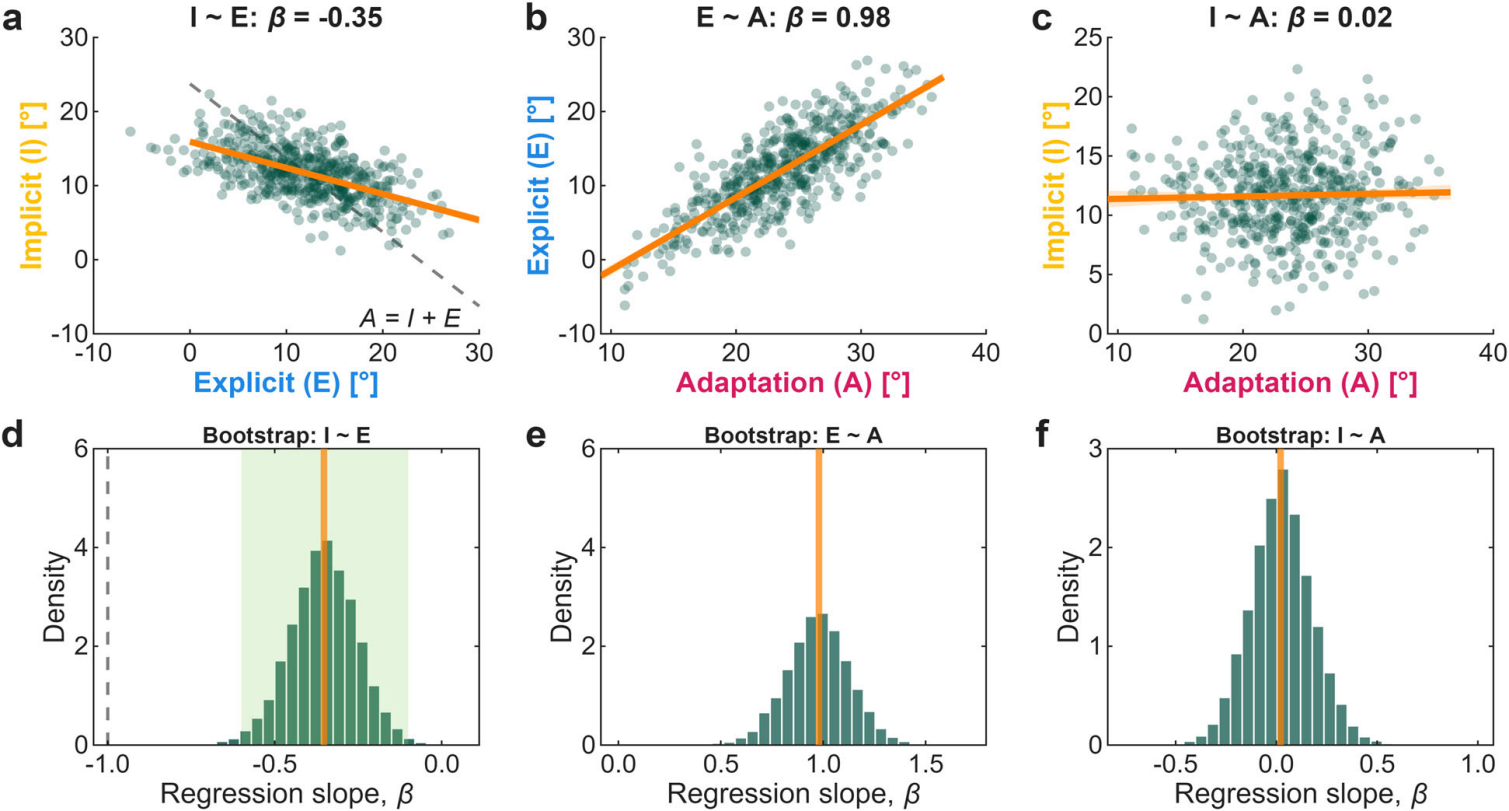
The shared-error model produces “sub-additive” slopes under additivity. ***a–c***, Scatterplots show the pairwise relationships among implicit (*I*), explicit (*E*), and total adaptation (*A*) for a simulated population (*N* = 1,000) generated from the shared-error model in which *A* = *I* + *E*. The orange lines show regression fits; the dashed black line in (***a***) indicates the expected slope of *β* = −1 if total adaptation were constant across individuals. ***d–f***, Bootstrap distributions of regression slopes from 10,000 resamples (*n* = 25 per sample). The orange vertical lines indicate population estimates. The dashed black line in ***d*** marks *β* = −1 and the light green shading indicates the range of slopes reported by ‘t Hart et al. (2024) and Chen and Taylor (2025). The *I* − *E* slopes cluster well above −1, consistent with empirical reports, despite the generative model being additive.

The shared-error model reproduced all three predicted relationships (Fig. 4). The *I* − *E* slope was negative (*β* = −0.35), falling within the range reported by ‘t Hart et al. (2024) and Chen and Taylor (2025; Fig. 4*d*, light green shading). The *E* − *A* slope was strongly positive (*β* = 0.98), and the *I* − *A* slope was near zero (*β* = 0.02), consistent with the regression dilution expected from measurement noise. Critically, these patterns emerged from a model in which *A* = *I* + *E* holds for every simulated participant.

Furthermore, the magnitude of the reported “sub-additive” slopes is predicted by the observed variance ratios. The slope equals the correlation scaled by the implicit-explicit variance ratio. Negative correlations produce negative slopes, but when explicit strategies vary more than implicit recalibration (*σ*_E_ > *σ*_I_; Bond and Taylor, 2015; Miyamoto et al., 2020), the slope magnitude is attenuated. This is not a sign of “sub-additivity,” but an expected statistical signature of an additive system with shared-error coupling.

In summary, slopes shallower than −1 do not indicate that implicit and explicit measures violate additivity. They indicate that the learning processes covary negatively and differ in their variance, exactly as shared-error models predict. Our choice of model was not arbitrary as the shared-error framework was developed within the same research program that later interpreted shallow slopes as evidence against additivity (Albert et al., 2022; ‘t Hart et al., 2024). The model assumes *A* = *I* + *E* throughout yet predicts the exact patterns claimed to falsify additivity.

## Discussion

The additivity assumption—implicit and explicit processes sum to produce motor output—has served as a foundation for sensorimotor adaptation for several decades. It justifies the use of subtractive measurement, informs computational models, and shapes how dissociations between learning processes are interpreted. Recently, this assumption has come under scrutiny. ‘t Hart et al. (2024) and Chen and Taylor (2025) report regression slopes significantly different from −1 and conclude that additivity is violated. If correct, this would require a substantial reconceptualization of how learning processes combine.

We argue that this conclusion is not supported by the evidence. This is not because additivity is necessarily true, but because regression slopes provide no evidence against it. Regression slopes quantify covariance across individuals, whereas additivity is a claim about combination within individuals: whether learning processes sum to produce observed behavior.

Under subtractive logic, the *β* = −1 benchmark holds only when total adaptation and the measured component (*I* or *E*) are uncorrelated, in which additivity does not require. Under independent measurement, the slope reflects the covariance structure of the learning processes, in which additivity also does not constrain. Moreover, the term “sub-additive” conflates covariance with combination. A slope shallower than −1 indicates negative correlation together with unequal variances. Thus, the slopes reported in the literature are fully consistent with existing additive models of motor learning, including those developed by some of the same authors (Albert et al., 2022).

### Additivity is about expression, not acquisition

The confusion in the literature stems from conflating acquisition and expression: how learning processes acquire their values and how those values are combined. Additivity is about expression. On any given trial, whatever values *I* and *E* have reached, they sum to produce motor output: *A* = *I* + *E*. This is about the readout, not the update.

Shared-error coupling describes acquisition. In shared-error models (Albert et al., 2022), implicit learning responds to residual error after explicit strategy is accounted for. The same basic formalism also predicts compensation when explicit strategy is noisy (Miyamoto et al., 2020): trial-to-trial noise in *E* enters the residual error signal and is tracked (with a lag and low-pass filtering) by the implicit process, yielding negative covariation between *I* and *E* even though the readout remains additive. In both cases, these effects reflect how the learning processes are driven during acquisition, not evidence against additive expression.

Learning processes can therefore interact during acquisition yet sum at expression. Consider two participants: one relies heavily on explicit strategy, leaving little residual error for implicit learning; the other uses minimal strategy, leaving large errors that drive substantial implicit recalibration. Across these individuals, *I* and *E* are negatively correlated, but within an individual, *A* = *I* + *E* could hold exactly.

In statistical terms, “interaction” often implies a multiplicative term in the output function (*I* × *E*). That is not what shared-error coupling or compensation describes. The interaction is in the update rule, not the combination rule. Whether the learning systems share error signals or compensate for noise, they can still sum linearly at expression. Thus, the “sub-additive” slopes reported by ‘t Hart et al. (2024) and Chen and Taylor (2025) are entirely compatible with additive shared-error models.

### Objection: task constraints

A natural objection is that the task itself enforces a tradeoff between implicit and explicit learning. If a participant must counteract a 30° rotation, then successful performance requires *I* + *E* = 30°. Under this constraint, any increase in explicit strategy must be matched by a corresponding decrease in implicit recalibration, mandating *ρ*_I,E_ = −1. This is the logic employed by ‘t Hart et al. (2024). If true, a shallower slope might suggest that the learning processes fail to sum.

But this argument assumes that all participants compensate for the perturbation to the same degree. They do not. Empirical data from adaptation studies show substantial individual variation: some participants under-compensate, others over-compensate, and others drift over time. This variance breaks the forced tradeoff between implicit and explicit learning. When total adaptation varies, the components need not trade off perfectly (Albert et al., 2022).

The argument also conflates the intended goal with the observed outcome. The task may demand 30°, but it does not guarantee it. Participants attempt to eliminate performance error, but the extent to which they succeed depends on learning rates (Smith et al., 2006; Albert et al., 2021; Wang et al., 2024), cognitive traits like working memory (Christou et al., 2016; O’Bryan et al., 2025), cognitive states like attention (Taylor and Thoroughman, 2007; Im et al., 2022), age (Vachon et al., 2020; Cisneros et al., 2024), disease status (Tseng et al., 2007; Butcher et al., 2017), and other sources of individual variability. Additivity says that whatever values *I* and *E* take, they sum to produce *A*. It does not say that *A* must equal the perturbation magnitude, nor does it constrain how *I* and *E* covary across individuals who achieve different levels of performance.

### Objection: measurement issues

A second objection concerns the mismatch between different measures of explicit learning. There has been substantial effort dedicated to highlighting the inconsistencies among operationalizations of implicit and explicit learning (Hadjiosif and Krakauer, 2021; Maresch et al., 2021a,b; ‘t Hart et al., 2024). Indeed, ‘t Hart et al. (2024) validly point out that aim reports often disagree with inclusion−exclusion differences, sometimes substantially. If both purport to measure explicit strategy, should they not agree? And if they do not, does that challenge the additivity assumption?

This concern is legitimate but misdirected. Discrepancies between measures primarily reflect validity problems, not necessarily non-additivity. Aim reports capture intended aiming direction before movement. Inclusion–exclusion differences capture strategy expression at retrieval, under task instructions that require participants to voluntarily engage or suppress a strategy they may not fully access consciously. The fact that these measures sometimes disagree tell us that our tools are imperfect, not that summation fails at the level of motor output.

Even with independent measures, learning processes are not temporally stable, so measurements taken at different times using different methods may not agree (Hadjiosif and Krakauer, 2021; Maresch et al., 2021a,b). Whether existing measures validly reflect implicit and explicit contributions is a psychometric question; whether those contributions sum is a compositional one. These problems also create attenuation bias. Because both implicit and explicit measures contain noise, measurement error attenuates the regression slope toward zero regardless of the true relationship.

### Objection: loose additivity

‘t Hart et al. (2024) also tested “loose additivity,” whether the sum of implicit and explicit measures predicts total adaptation with a slope of 1 and intercept of 0. This is closer to a direct test: if *A* = *I* + *E*, then regressing *A* on (*I* + *E*) should yield the identity line. They reported mixed results, with some groups supporting loose additivity and others not.

This test is more appropriate than the *I* ∼ *E* regression, but it remains vulnerable to measurement error. When both *I* and *E* contain noise, their sum is a noisy predictor. Error in the independent variable dilutes regression slopes toward zero. A slope less than 1 therefore does not imply that the processes are non-additive; it suggests instead that our measurements, and perhaps even our constructs, are imperfect (Hadjiosif and Krakauer, 2021; Maresch et al., 2021a,b).

**Figure 5.**
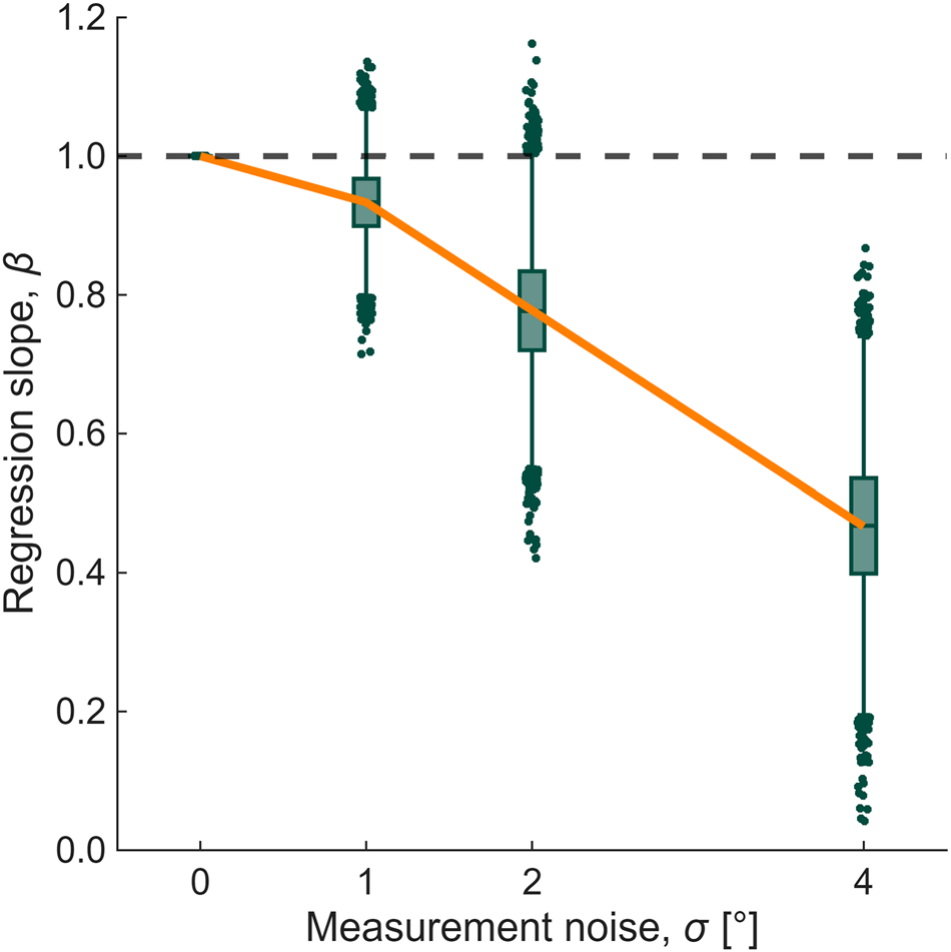
Measurement noise undermines the “loose additivity” benchmark. Data were simulated under an additive model (*A* = *I* + *E*) with correlated implicit and explicit processes (*ρ* = −0.5). Independent Gaussian noise was added to *I* and *E* before their sum was computed. Regressing *A* on (*I*_obs_ + *E*_obs_) yields slopes progressively below 1 as measurement noise increases, despite the generative model being additive. The orange line shows the predicted attenuation; boxplots show bootstrap distributions (*n* = 25, 10,000 iterations). At realistic noise levels (*σ* = 2–4°), the estimated slopes fall well below 1.

This attenuation is predictable. If implicit and explicit measures each have imperfect reliability, the composite (*I* + *E*) inherits that noise: Var(*I* + *E*) = Var(*I*) + Var(*E*) + 2Cov(*I*,*E*). Under classical measurement theory, the regression slope is diluted by the reliability of the predictor. With realistic noise levels for aim reports and exclusion trials, slopes below 1 are expected even under perfect additivity (Fig. 5). The partial support for “loose additivity” reported by ‘t Hart et al. (2024) is therefore consistent with noisy measurement.

Even if measurement precision were sufficient, interpreting deviations from *A* = *I* + *E* would remain ambiguous. Sub-additivity could indicate that a process is missing from the model—perhaps the true system is *A* = *I* + *E* + *X*, where *X* is unmeasured. Alternatively, it could indicate that *I* and *E* genuinely interact at output. A missing additive process and genuine non-additivity can produce the same empirical pattern.

Figure 1, *d*–*f*, illustrates this ambiguity with an analogy to fluid mixing. If implicit and explicit processes combine like non-mixing fluids, their volumes should sum exactly. If they combine like water and ethanol, volume contracts slightly upon mixing, and detecting that contraction requires precise measurement. The modest deviations reported in the literature are therefore consistent with either ideal mixing plus measurement noise or mild non-ideal mixing obscured by that same noise. Regression slopes cannot distinguish these possibilities because they measure covariance across individuals, not combination within them.

### Toward better tests of additivity

‘t Hart et al. (2024) and Chen and Taylor (2025) raise an important question: should sensorimotor adaptation continue to use additivity as a working assumption? This scrutiny is appropriate and necessary. The assumption is consequential as it justifies subtractive measurement, shapes computational models, and influences how interactions between learning systems are interpreted. If it is wrong, we want to know sooner rather than later.

The problem is that regression slopes cannot answer this question. Residual analysis—computing *ε* = *A* − *I* − *E* and assessing whether it is near zero—is conceptually correct but difficult. The three components are never measured under identical conditions or even at the same time, so non-zero residuals confound genuine non-additivity with temporal decay of implicit learning, measurement reactivity from the act of aim reporting, and methodological biases whereby different measures capture different strategic components (Hadjiosif and Krakauer, 2021; Maresch et al., 2021a,b).

Although non-additive models have not, to our knowledge, been explicitly proposed, we can suggest the kinds of residual structure they would predict. An additive model predicts that residuals (*A* − *I* − *E*) should be centered on zero and uncorrelated with *I*, *E*, or their product. A multiplicative gating model would instead predict residuals that covary systematically with *E*. A saturating model would predict residuals that grow more negative as *I* + *E* increases. Each alternative leaves a distinct fingerprint in the residual structure. Regression slopes cannot detect these patterns, but precise residual analysis could.

What would count as evidence against additivity? Systematically biased residuals that covary with *I* or *E* in ways consistent with multiplicative gating or saturation and that cannot be attributed to known measurement problems. This is a high bar, and appropriately so. The additivity assumption is deeply embedded in sensorimotor learning, and overturning it requires more than testing regression slopes against arbitrary benchmarks. Formal model comparison offers a better path forward: specify competing generative models and compare their ability to predict empirical data. This shifts the debate away from ambiguous regression slopes and toward parsimony, predictive adequacy, and mechanistic interpretability.

## Data Availability

All simulation and analysis code is freely available online at https://doi.org/10.5281/zenodo.19961504. Simulations were conducted in MATLAB 2025b on a Windows 11 OS. The code is self-contained and will generate all figures in the paper.

## Synthesis

Reviewing Editor: David Schoppik, New York University Grossman School of Medicine Decisions are customarily a result of the Reviewing Editor and the peer reviewers coming together and discussing their recommendations until a consensus is reached. When revisions are invited, a fact-based synthesis statement explaining their decision and outlining what is needed to prepare a revision will be listed below. The following reviewer(s) agreed to reveal their identity: Alkis Hadjiosif.

We found the paper well-written, especially in its step-by-step explanation of the underlying statistical issues. The finding is useful to the field, as it corrects a misunderstanding with real implications in our understanding of motor learning (whether explicit and implicit learning add perfectly or not) and how we should measure it. It is important to note that this work does *not* prove that sub-additivity is not the case; it shows that the slope test used to assess sub-additivity is not the right one.

We did not find any substantial issues with this work. Thank you for your patience with a longer-than-usual review process.

We note that Figure 5 and related analysis seem more like Results than Discussion, though they do fit with the Discussion narrative.

Potential minor errors in Figure 4 d-f:

In 4d, the x-tick-label -1.0 looks wrong, as -1.0 is the vertical dashed line and the x-tick is to the left of it

In 4f, there seems to be an error: the x-ticks are equidistant (step=0.5); but the label below the first and second x-ticks are -0.8 and -0.4, i.e. step=0.4. It seems the first x-label should be -0.9

A mild complaint: it is uncommon (and a bit hard to read) when x-ticks bypass zero (e.g. going from -0.4 to 0.1). Please mark zero.

## Author Response

Thank you for the positive assessment of the manuscript and for the constructive feedback from the reviewers. Below I address each point raised in the synthesis statement.

Comment 1: Figure 5 placement. We note that Figure 5 and related analysis seem more like Results than Discussion, though they do fit with the Discussion narrative.

Response: Thank you for this observation. Because Figure 5 demonstrates a conceptual point about measurement noise, and because the reviewers agreed it fits the Discussion narrative, I kept it in its current location rather than creating a new section.

Comment 2: Figure 4d x-tick label misalignment. The x-tick-label -1.0 looks wrong, as -1.0 is the vertical dashed line and the x-tick is to the left of it.

Response: Thank you for identifying this mistake. The auto-generated tick labels have been replaced with manually specified values that include zero and use clean, evenly spaced intervals (e.g., -1.0, -0.5, 0.0). The tick labels now align correctly with their tick marks and with the vertical reference line at the true slope.

Comment 3: Figure 4f x-tick spacing error. There seems to be an error: the x-ticks are equidistant (step=0.5); but the label below the first and second x-ticks are -0.8 and -0.4, i.e. step=0.4. It seems the first x-label should be -0.9.

Response: The same manual tick-labeling approach was applied to Figure 4f, ensuring consistent spacing and correct alignment between tick marks and labels.

Comment 4: x-axes bypassing zero. A mild complaint: it is uncommon (and a bit hard to read) when x-ticks bypass zero (e.g. going from -0.4 to 0.1). Please mark zero.

Response: I completely agree. It is difficult to orient to the axis values without zero marked as a reference point. Apologies for the use of the auto-generated labels.

Minor editorial revisions were made throughout to improve clarity, reduce repetition, sharpen section endings, and tighten conceptual distinctions without changing the manuscript's substantive claims. I also revised the figures to use a colorblind-friendly palette. Thank you again for the opportunity to revise this manuscript. I believe these changes address all concerns raised by the reviewers and editor.
